# Identifying Family Relationships from Electronic Health Records: A Machine Learning Approach

**DOI:** 10.64898/2026.09.18.26363428

**Published:** 2026-09-21

**Authors:** Abhinav Pundir, Andrew Hill, Michael G. Kahn, Bethany M. Kwan, Daniel M. Lindberg, Shaun J. Grannis, Titus K. Schleyer, Lisa M. Schilling, Sarah V. Kautz, Toan C. Ong

## Abstract

**Objective:**

To develop and evaluate machine learning methods for automated identification of family relationships from electronic health records (EHRs). We employed Random Forest classifiers to identify five relationship types: Mother-Child, Father-Child, Sibling-Sibling, Twin-Twin, and Partner-Partner, using a four-stage iterative refinement process to improve precision for patient-centered outcomes research (PCOR) and healthcare applications.

**Materials and Methods:**

We used two large-scale Indiana datasets: the Indiana Network for Patient Care (INPC), comprising approximately 15 million unique individuals across approximately 45 million medical records, and the Indiana Natality dataset (birth certificates, 1970-2024), which served as the gold standard. Positive cases were derived by linking verified relationships from Natality records to INPC using a shared Global Identifier. Negative cases were drawn from a three-tier blocking strategy combined with sliding window restriction and similarity scoring, which together reduced the comparison space from over 10 ^14^ potential pairs to approximately 121 million candidates. Five Random Forest classifiers underwent four-stage iterative refinement: initial training, feature removal for multicollinearity, feature engineering to address systematic errors, and training-data refinement restricted to relationship pairs with at least one shared contact feature.

**Results:**

The models achieved precision of 0.92 to 1.00, recall of 0.97 to 1.00, and F1 scores of 0.94 to 1.00 across all relationship types (Mother-Child 0.97, Father-Child 0.98, Sibling-Sibling 0.98, Twin-Twin 1.00, Partner-Partner 0.94). Between 15% and 78% of Natality-verified relationships lacked any shared contact information in INPC and were structurally undetectable by record linkage; restricting training and evaluation to linkable pairs improved precision, and training-data refinement improved F1-scores by a further 0.02-0.04 for the Mother-Child, Father-Child, and Sibling models. High-confidence predictions (probability ≥ 0.9) captured 77-99% of true positives. Age difference was the primary predictor for parent-child and twin relationships, while phone number similarity was most important for siblings.

**Discussion:**

The framework identifies family relationships from standard demographic fields available in most EHR systems. The iterative refinement approach shows how systematic error analysis can guide methodological improvements. Training-data refinement addresses a constraint in EHR-based family linkage: not all verified biological relationships have overlapping demographic footprints in healthcare data. Unlike rule-based approaches, the framework provides probability scores that enable configurable deployment thresholds.

**Conclusion:**

The Random Forest models demonstrate high performance suitable for large-scale research deployment and are ready for testing in clinical applications rather than immediate widespread clinical use. The methodology can be adapted to other EHR systems, supporting family-centered study design in patient-centered outcomes research.

## Full Text Availability

The license terms selected by the author(s) for this preprint version do not permit archiving in PMC. The full text is available from the preprint server.

